# Supplementary material for: Chromothripsis during telomere crisis is independent of NHEJ, and consistent with a replicative origin
Source: Genome Res. 2019 May;29(5):737–49. doi: 10.1101/gr.240705.118 (PMC6499312; doi:10.1101/gr.240705.118)
Supplement: Supplemental Material [file supp_gr.240705.118_Supplemental_file_1.zip › contigs/annotated_contigs/DB51/contig.2.DB51_length_277_mean_cov_7.03971119134.docx]

**DB51_length_277_mean_cov_7.03971119134**

```
AAGAGTGACTTTCCTAGAACTGAGGTGTAGCTTAATCTATATGTACAAATCTAATAAAGTATGTCACTTGAGACTTAAACATAGCCAAT
 >chr6:70380161-70380331 + E=3e-91
ATATGTGTTGAGAACTGTCCAAGGCAGCACAACTCAATCCGGGCAACTTGGGCAATCTGGTCCCCTTAGAGTGGTCTCT|GC|ACTCAT
                                                                                >chr6:6
GCCATTCTACAAGTAGATTTTTATAGCATTTCTTCATACATATAAATATTACATAAAAGACCATTACATTTATTAAAATGCCAAGTACT
6658365-66658474 + E=3e-51
TTTATTTGTACT
```
